# Vitamin K deficiency bleeding presenting as impending brain herniation

**DOI:** 10.4103/1817-1745.66681

**Published:** 2010

**Authors:** H. Gopakumar, R. Sivji, P. K. Rajiv

**Affiliations:** Department of Neonatology, Amrita Institute of Medical Sciences (AIMS), Kochi, Kerala, India

**Keywords:** Intracranial hemorrhage, vitamin K deficiency bleeding

## Abstract

It is presently a universal practice to administer vitamin K at birth. Hence, the serious bleeding manifestations from vitamin K deficiency are nowadays very rare. We describe a case of late vitamin K deficiency bleeding presenting as intracranial hemorrhage with impending coning and the related review of literature. Such severe bleeding episodes due to vitamin K deficiency are associated with multiple cranial involvement and impending brain herniation is probably rare.

## Introduction

A 2-month-old male child was admitted with history of excessive cry and lethargy of two days and poor feeding of one day duration. Baby also had abnormal posturing of limbs and asymmetry of eye opening since one day. Parents had noticed progressive pallor over the previous two days. They attributed the complaints to a history of insect bite on thighs two days back for which the child received supportive treatment from a nearby hospital. There was no history of fever or head trauma. There was no significant past illness.

In view of abnormal posturing and progressive pallor, baby was referred to our hospital. Baby was born to a primi mother by emergency LSCS, indication being cephalopelvic disproportion. Baby cried soon after birth. Immediate postnatal period was uneventful. Baby was exclusively breast fed from birth. Mother was not sure whether vitamin K was administered at birth.

At the time of admission in our hospital, baby had irritable cry with intermittent decorticate posturing. He had severe pallor. There were no obvious bleeding manifestations. There was no icterus. Anterior fontanel was bulging and non pulsatile. There were frequent episodes of bradycardia with high blood pressure and irregular respiration. Capillary refill time was less than 2 sec. Detailed neurological examination revealed facial asymmetry, right sided ptosis with ipsilateral dilated pupils. Findings were consistent with right sided third nerve palsy with uppermotor neuron facial palsy. Tone was increased in all four limbs with exaggerated deep tendon reflexes.

Blood investigations showed unduly prolonged PT, APTT with no detectable clot. Other blood investigation showed hemoglobin value of 4.62 gm%, Hematocrit – 12.5%, total count – 21, 000 / cmm, C Reactive protein – 15 mg / l, Serum creatinine – 0.43 mg / dl, SGPT – 29.6 mg / dl, SGOT – 48.8 mg / dl, alkaline phosphatase – 408. 3, serum bilirubin – 1.6 mg / dl (direct – 0.8 mg / dl), serum albumin – 4.3 gm/dl, serum globulin – 2.3 gm/dl, protein – 6.6 gm/ dl, serum sodium – 133 meq / dl, serum potassium – 5.1meq/dl plasma urea – 32 mg / dl, serum calcium – 9.3 mg / dl, platelet count – 7.11 lakhs / cmm, differential count (neutrophills – 54.9%, lymphocytes – 37.1, monocytes – 6.71%, eosinophills – 0.013% and basophils – 1.25%) plasma fibrinogen activity – 387

In view of prolonged prothrombin time and APTT in an otherwise well child, the possibility of late onset vitamin K deficiency bleeding was considered. He was administered 15 ml / kg of fresh frozen plasma and vitamin K with which PT, APTT and INR improved dramatically (13.6 / 14.6, 26.6 / 32.2 and 0.92 respectively), further confirming our diagnosis. Packed cell transfusion was also given in view of clinically significant anemia.

**Figure 1:**
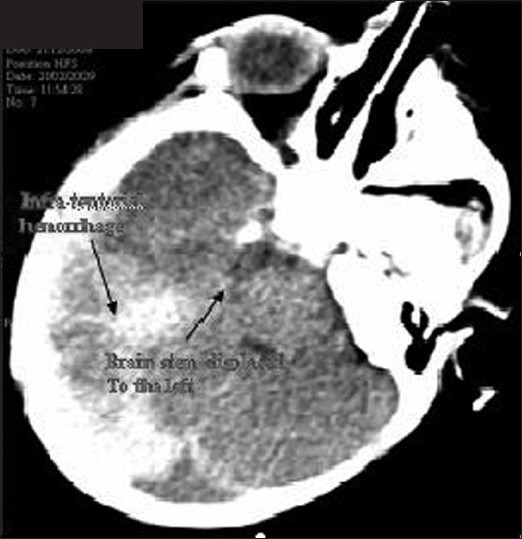
Infratentorial bleed with brainstem displaced

**Figure 2:**
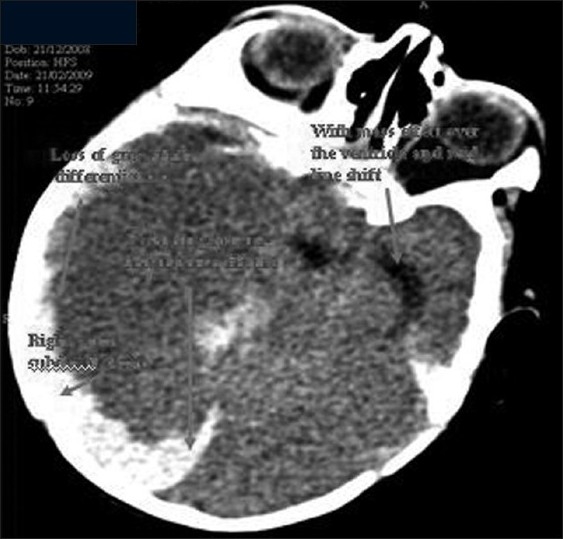
Subdural bleed and gross midline shift

Initial CT scan [Figures 1 and 2] showed extensive acute subdural hemorrhage involving the right convexity (frontoparieto occipital region). Blood fluid level was noted in the right frontal subdural bleed, suggestive of acute on chronic bleed. Subdural hemorrhage was also noted along the inter hemisphere fissure. Right tentorial subdural hemorrhage was also seen. Subdural hemorrhage was also noted in left frontal region with blood fluid level. The right lateral ventricle was completely effaced with midline shift of 1.02 cm. The suprasellar and ambient cistern were effaced, suggestive of bilateral uncal herniation.

A diagnosis of late onset vitamin K deficiency causing bilateral subdural hemorrhage with attendant uncal herniation clinically manifested as right third nerve palsy and left hemiparesis was made. Subdural hematoma was more on right than left.

Seizures were controlled with phenobarbitone, phenytoin and midazolam. Baby was electively intubated and measures to decrease intracranial pressure were given.

Baby was taken up for emergency drainage of subdural bleed under cover of mannitol. A right Frontotemporoparietal craniotomy and evacuation of SDH with lax duroplasty was done. Peroperative findings included very tight brain, with various ages of clot. Brain was bulging out of the craniotomy due to excess tension. Pulsation was very feeble. Sub dural membrane was adherent with dura mater and brain surface in few areas. A left extraventricular drainage was also placed.

Underlying brain was found to be friable. External ventricular drainage with intracranial pressure monitoring was continued postoperatively. The preoperative findings suggested that baby had minor episodes of bleeding previously as well, which was not severe enough to cause clinical manifestations.

In the initial 48 h, intracranial pressure was very high with continuous draining of CSF through external ventricular drainage. Child also had uncontrolled focal seizures that required midazolam infusion and muscle relaxants.

**Figure 3:**
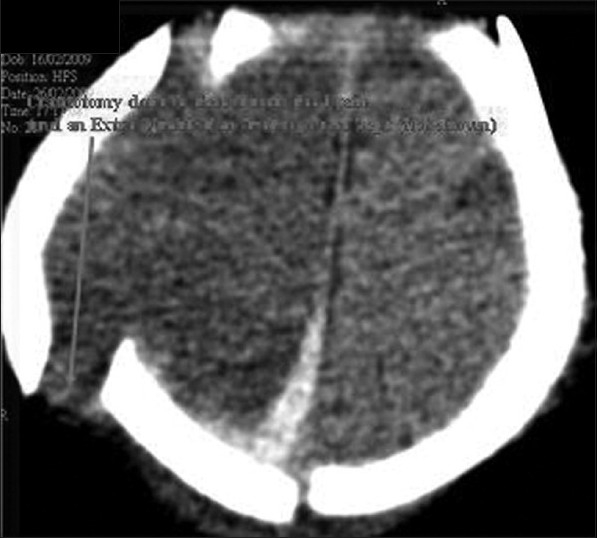
Post craniotomy

After 48 h, intracranial pressure came down. External ventricular drainage was minimal. Baby was free of seizures, right ptosis improved and pupils were equal and reactive bilaterally. His conscious level improved. Neurosonogram was repeated which showed no midline shift. Repeated CT scan [Figure 3] showed small amount of persisting subdural bleed. There was no midline shift. PT and APTT repeated twice were reported normal.

Baby was extubated on sixth postoperative day. External ventricular drainage was removed. Feeds was started and increased as per tolerance to full feeds. Baby was discharged on fourteenth postoperative day. At the time of discharge, baby was active and feeding well. Pupils were equal and reactive bilaterally. There was mild facial asymmetry with left sided hemiparesis. There were no further episodes of seizures. Baby is kept on regular neurodevelopmental follow up.

## Discussion

Vitamin K is a fat soluble vitamin essential for the synthesis of functional prothrombin, factor VII, factor IX and factor X in the liver. Because of the short half-life of these factors, and the small amounts of vitamin K that can be stored in the body, inadequate intake of vitamin K can result in deficiency in a short period of time. PIVKA, inactive precursor proteins induced in vitamin K absence, are measurable and can be used as an indicator of vitamin K deficiency.

Vitamin K deficiency can occur in persons of any age. Infants are at higher risk for hemorrhagic disease of newborn, caused by a lack of vitamin K reaching the fetus across the placenta, the low level of vitamin K levels in breast milk, immature liver and low colonic bacterial synthesis.

The bleeding manifestations associated with vitamin K deficiency can occur in three general time frames. Early onset vitamin K deficiency bleeding that occurs less than 24 h after birth is rare and is almost always associated with maternal medications that interfere with vitamin K metabolism. The various drugs include anticonvulsants, anticoagulants, and antibiotics. Postnatal administration of vitamin K has no effect in preventing early-onset disease. Vitamin K supplementation to at risk mothers, administered prenatally may prevent this form of vitamin K deficiency bleeding.

The classic onset of vitamin K deficiency bleeding occurs between second and seventh day after birth in breastfed infants. Clinical manifestations include bleeding in to the skin or from mucosal surfaces, circumcision, or venipuncture sites.[1]

Late-onset vitamin K deficiency bleeding occurs two weeks after birth. It can, however, occur as late as 3 months postpartum. In addition to breastfeeding, risk factors include diarrhea, hepatitis, cystic fibrosis (CF), celiac disease, and alpha - 1-antitrypin deficiency or absence of prophylaxis in otherwise healthy infants. Late-onset vitamin K deficiency bleeding tends to be more severe than early-onset or classic disease and has a high frequency of intracranial hemorrhage. Intracranial hemorrhage is observed in more than 50% of infants with late-onset disease.

Almost all neonates are vitamin K deficient, presumably as a result of deficient vitamin K nutrition in the pregnant mother during the third trimester and because of the lack of colonization of the colon by bacteria that produce vitamin K in the neonate.[2] However, this deficiency is further aggravated in some patients by inadequate dietary intake of vitamin K. This disorder is more prevalent in breast-fed babies, as human milk, in contrast to cow’s milk, contains only 15 μg/l of vitamin K.[3]

The risk of vitamin K deficiency bleeding in babies whose mothers are exposed to anticonvulsants during pregnancy appears to be because of competitive inhibition of the synthesis of the calcium-binding gamma-carboxyglutamic acid residues to the precursors of the clotting factors II, VII, IX, and X and by the induction of fetal microsomal enzymes, which degrade vitamin K. The pattern of the bleeding in anticonvulsant-exposed newborn infants differs from hemorrhagic disease of unexposed infants: the bleeding occurs earlier, often within the first hours of life; the extent of the hemorrhage is greater; the sites of the bleeding are different and include intrabdominal, intracranial, and intrathoracic locations.[4] Supplementation with vitamin K, 10 mg/day during the last month of pregnancy, has been recommended by the American Academy of Neurology.[5] Other drugs implicated to cause the deficiency in newborns include maternal intake of oral anticoagulants and large doses of aspirin.

Malabsorption syndromes are commonly associated with vitamin K deficiency. Defects in the enterohepatic circulation because of biliary disease interfere with absorption of fat-soluble vitamins in the ileum. Primary biliary cirrhosis, cholestatic hepatitis, and other causes of cholestasis can lead to impaired absorption of vitamin K. Furthermore, intestinal malabsorption, as in sprue or regional enteritis, impairs vitamin K use. Older adults also have evidence of mild vitamin K deficiency, presumably because of intestinal malabsorption.[6]

Bacteria in the large intestine produce functional forms of vitamin K. In the absence of dietary vitamin K, small amounts of vitamin K in the large intestine are absorbed passively and prevent severe vitamin K deficiency. This source is eliminated in patients who are on antibiotics, that deplete the intestinal flora capable of vitamin K synthesis. Thus, a common setting of vitamin K deficiency is the case of a patient with inadequate or minimal dietary intake, who is also on treatment with antibiotics.

This form of vitamin K deficiency occurs within 1 to 3 weeks, after depletion of body stores of vitamin K.

Neonates with vitamin K deficiency have a prolonged prothrombin time and partial thromboplastin time (PTT). However, it is critical to distinguish whether the prolongation of these parameters is a manifestation of the deficiency of the vitamin K-dependent proteins because of vitamin K deficiency or to decreased synthetic capacity of the liver in newborns. Elevation of the abnormal (des-γ-carboxy) prothrombin (PIVKA-II) antigen level is indicative of vitamin K deficiency, as this form of prothrombin appears only when post-translational modification is impaired but not when protein synthesis is impaired. We could not do this test in our baby due to lack of facility for the same. Liver function tests done to rule out any liver pathology causing vitamin K deficiency was reported negative.

A confirmed case of VKDB should fulfill the diagnostic criteria of having a PT that is >4 the control value and display at least one of the following: (1) Normal or raised platelet count, normal fibrinogen and absent fibrin degradation products. (2) PT returning to normal after VK administration. (3) PIVKA (usually that of factor II) level exceeding normal controls. A probable case of VKDB is defined as one in which the PT and APTT are abnormal for age and one of the three extra criteria listed above is present.[7] Recently, the British Paediatric Surveillance Unit (BPSU) for their current survey (October 2006-2008) have provided a simplified definition for vitamin K deficiency bleeding ‘Any infant under six months of age with spontaneous bruising/bleeding or ICH associated with prolonged clotting times (PT at least twice control value) and normal or raised platelet count, NOT due to an inherited coagulopathy or disseminated intravascular coagulation’. Our case satisfies this definition of prolonged prothrombin time along with a raised platelet count.

Administration of vitamin K (100 μg) corrects the deficiency state and usually does not need to be repeated in the otherwise healthy infant. In our patient, a single injection of vitamin K normalized the coagulation parameters. In practice, deficiency of vitamin K is confirmed by showing that the prolonged prothrombin time is rapidly corrected in 6 to 24 h after parenteral administration of Vitamin K

Vitamin K (100 μg to 1 mg) is administered intramuscularly to the newborn immediately after birth. At these doses, vitamin K administration carries little morbidity and can prevent hemorrhagic disease of the newborn.

The approach to the treatment of vitamin K deficiency depends on the clinical setting and the severity of bleeding. Except in the face of serious internal bleeding, reversal of the vitamin K deficiency by the administration of vitamin K is generally adequate. If there is no hepatic dysfunction, its administration is followed by an increase in prothrombin time above the minimal level required for hemostasis within 3 to 4 h, and usually a return to normal in about 24 h. The response to treatment should be determined in all cases by repeating the prothrombin time test 24 h after commencement of treatment.

If the prothrombin time (PT) is significantly prolonged to indicate that a bleeding complication may be induced by intramuscular injection, this route should be avoided. Because the delivery of vitamin K by the subcutaneous route is variable, intravenous administration of vitamin K_1_ is the recommended approach, as it ensures rapid delivery. However, intravenous administration of vitamin K1 does require monitoring, because of certain reports of severe allergic reactions. Care must be given to initiate rapid reversal of an untoward reaction.

Serious bleeding complications attributed to vitamin K deficiency, such as intracranial bleeding, must be reversed immediately. Despite the rapid action of vitamin K, administration of vitamin K should be preceded by the infusion of fresh frozen plasma. This blood component contains all the vitamin K-dependent clotting proteins. In sufficient quantities, fresh frozen plasma can correct, or nearly correct, the PT, as well as the bleeding tendency.

Patients with vitamin K deficiency without bleeding manifestations can be treated with oral vitamin K or, as in patients with chronic vitamin K deficiency secondary to malabsorption syndromes, with subcutaneous vitamin K.
